# Inhibition of Crmp1 Phosphorylation at Ser522 Ameliorates Motor Function and Neuronal Pathology in Amyotrophic Lateral Sclerosis Model Mice

**DOI:** 10.1523/ENEURO.0133-22.2022

**Published:** 2022-05-23

**Authors:** Tetsuya Asano, Haruko Nakamura, Yuko Kawamoto, Mikiko Tada, Yayoi Kimura, Hiroshi Takano, Ryoji Yao, Hiroya Saito, Takuya Ikeda, Hiroyasu Komiya, Shun Kubota, Shunta Hashiguchi, Keita Takahashi, Misako Kunii, Kenichi Tanaka, Yoshio Goshima, Fumio Nakamura, Hideyuki Takeuchi, Hiroshi Doi, Fumiaki Tanaka

**Affiliations:** 1 Department of Neurology and Stroke Medicine, Yokohama City University Graduate School of Medicine, Yokohama 236-0004, Japan; 2 Advanced Medical Research Center, Yokohama City University, Yokohama 236-0004, Japan; 3 Department of Cell Biology, Cancer Institute, Japanese Foundation for Cancer Research, Tokyo 135-8550, Japan; 4 Department of Molecular Pharmacology and Neurobiology, Yokohama City University Graduate School of Medicine, Yokohama 236-0004, Japan; 5 Department of Biochemistry, School of Medicine, Tokyo Women’s Medical University, Tokyo 162-8666, Japan

**Keywords:** ALS, CRMP1, SOD1

## Abstract

Amyotrophic lateral sclerosis (ALS) is a rapidly progressive and fatal neurodegenerative disorder that affects upper and lower motor neurons; however, its pathomechanism has not been fully elucidated. Using a comprehensive phosphoproteomic approach, we have identified elevated phosphorylation of Collapsin response mediator protein 1 (Crmp1) at serine 522 in the lumbar spinal cord of ALS model mice overexpressing a human superoxide dismutase mutant (SOD1^G93A^). We investigated the effects of Crmp1 phosphorylation and depletion in *SOD1^G93A^* mice using Crmp1^S522A^ (Ser522→Ala) knock-in (*Crmp1^k^*^i^*^/ki^*) mice in which the S522 phosphorylation site was abolished and *Crmp1* knock-out (*Crmp1*^−/−^) mice, respectively. *Crmp1^ki^*^/^*^ki^*/*SOD1^G93A^* mice showed longer latency to fall in a rotarod test while *Crmp1*^−/−^/*SOD1^G93A^* mice showed shorter latency compared with *SOD1^G93A^* mice. Survival was prolonged in *Crmp1^ki^*^/^*^ki^*/*SOD1^G93A^* mice but not in *Crmp1*^−/−^/*SOD1^G93A^* mice. In agreement with these phenotypic findings, residual motor neurons and innervated neuromuscular junctions (NMJs) were comparatively well-preserved in *Crmp1^ki^*^/^*^ki^*/*SOD1^G93A^* mice without affecting microglial and astroglial pathology. Pathway analysis of proteome alterations showed that the sirtuin signaling pathway had opposite effects in *Crmp1^ki^*^/^*^ki^*/*SOD1^G93A^* and *Crmp1*^−/−^/*SOD1^G93A^* mice. Our study indicates that modifying CRMP1 phosphorylation is a potential therapeutic strategy for ALS.

## Significance Statement

Collapsin response mediator protein 1 (CRMP1) is an intracellular molecule that mediates semaphorin 3A (Sema3A) signaling. Phosphoproteomic analysis showed that the semaphorin neuronal repulsive signaling pathway, which includes Crmp1 phosphorylation at Ser522, is upregulated in *SOD1^G93A^* mice that serve as a model of amyotrophic lateral sclerosis (ALS). While deleting both copies of the Crmp1 gene (*Crmp1*^−/−^) leads to deterioration of motor function in *SOD1^G93A^* mice, phospho-null Crmp1 (*Crmp1^ki/ki^*) improves motor function while preventing motor neuron loss and denervation of neuromuscular junctions (NMJs). Among the Sema3A-mediated axon guidance pathways, we propose that CRMP1 phosphorylation is a potential therapeutic target for ALS.

## Introduction

Amyotrophic lateral sclerosis (ALS) is a rapidly progressive and fatal neurodegenerative disorder that mainly affects motor neurons in the brain and spinal cord (Taylor et al., 2016). Only a few treatments with limited efficacy are currently available (Kim and Taylor, 2017). Since mutations in *SOD1*, which encodes superoxide dismutase 1 (SOD1), were first discovered to cause ALS in 1993 (Rosen et al., 1993), at least 27 genes have been found to be definitively associated with familial ALS, including transactive response DNA binding protein (*TARDBP*), fused in sarcoma (*FUS*) and chromosome 9 open reading frame 72 (*C9ORF72*; Chia et al., 2018).

Some of the pathogenic proteins responsible for neurodegenerative diseases have been shown to regulate and modulate diverse protein functions and intracellular pathways through posttranslational modifications such as phosphorylation, acetylation, and methylation. In spinocerebellar ataxia type 1 (SCA1), phosphorylation of ataxin-1, the causative protein for SCA1, plays a critical role in ataxin-1 aggregation (Emamian et al., 2003). Similarly, phosphorylation or acetylation of huntingtin alters its aggregation properties and neuronal toxicity in Huntington’s disease (HD; Arbez et al., 2017). In ALS, cytoplasmic aggregates of RNA-binding proteins such as phosphorylated TDP-43 (encoded by *TARDBP*) or FUS constitute a well-known, major pathologic marker for ALS. Acetylation of TDP-43 inhibits RNA binding and promotes the aggregation of phosphorylated TDP-43 (Cohen et al., 2015). By contrast, phosphorylation of FUS inhibits FUS aggregation and ameliorates FUS-related cytotoxicity, while loss of arginine methylation of FUS promotes FUS aggregation (Hofweber et al., 2018). Beyond the context of these proteins that directly cause disease, the importance of posttranslational modifications is evidenced by the finding that removing phosphorylation sites of neurofilament (NF) delays disease onset and prolongs survival in ALS model mice (Lobsiger et al., 2005). However, there is currently a limited understanding of how protein phosphorylation contributes to ALS pathogenesis. We therefore conducted phosphoproteomic analysis in an ALS model using the *SOD1^G93A^* mouse line, which is the most extensively used type of mouse in the study of ALS.

Through this analysis, we identified Collapsin response mediator protein 1 (Crmp1), previously not known to be highly phosphorylated in ALS model mice. CRMP1 belongs to a family of neuronal phosphoproteins (the CRMPs) and was originally identified as an intracellular protein that mediates semaphorin 3A (Sema3A) signaling (Goshima et al., 1995). CRMPs have been correlated with neurologic disorders such as Alzheimer’s disease (Uchida et al., 2005; Petratos et al., 2008; Ikezu et al., 2020), HD (Stroedicke et al., 2015), and schizophrenia (Yamashita et al., 2013; Nakamura et al., 2018; Nomoto et al., 2021). Additionally, Sema3A-CRMPs signaling has been suggested to be involved in ALS pathogenesis because Sema3A is upregulated in the motor cortex of ALS patients and the terminal Schwann cell adjacent to neuromuscular junctions (NMJs) in *SOD1^G93A^* mice (De Winter et al., 2006; Körner et al., 2016). Moreover, elevation of both Crmp4 and Crmp4-dynein complex leads to neuronal death in ALS model mice (Duplan et al., 2010; Maimon et al., 2021) while the inhibition of Crmp2 phosphorylation ameliorates the motor phenotype of *SOD1^G93A^* mice (Numata-Uematsu et al., 2019). However, it remains uncertain whether CRMP1 is involved in the pathomechanism of ALS. The only available data on CRMP1 in ALS is that Crmp1 and Crmp4 are highly abundant in the interactome of M337V mutant compared with wild-type (WT) TDP-43 (Feneberg et al., 2020). CRMP1 regulates neuronal cell migration, dendritic spine development, and synaptic plasticity through CRMP1 phosphorylation. CRMP1 and CRMP2 are phosphorylated by cyclin-dependent kinase 5 (Cdk5) at Ser522 (Cole et al., 2006; Yamashita et al., 2007). This phosphorylation is essential for mediating intracellular Sema3A signaling and primes the subsequent phosphorylation of Thr509, Thr514, and Ser518 residues by glycogen synthase kinase 3β (GSK3β or Gsk3b in mouse; Uchida et al., 2005; Cole et al., 2006). CRMP1 is also phosphorylated by Fyn at Tyr504 but not Ser522 (Kawashima et al., 2021). In this study, we investigated the effects of Crmp1 on disease progression in an ALS mouse model, and found that Crmp1 phosphorylation at Ser522 is a key event in motor impairment in ALS.

## Materials and Methods

### Ethics statement

This study was conducted in strict accordance with the Yokohama City University Guide for the Care and Use of Laboratory Animals (permission numbers F-A-19-030 and F-A-16-069), and experimental protocols were approved by the Independent Review Boards of Yokohama City University (permission numbers F-D-21-49 and F-D-18-70).

### Animal

C57BL/6N mice for producing zygotes and MCH-ICR mice to act as recipient and foster mothers were purchased from CLEA Japan.

### Generation of *Crmp1^ki^*^/^*^ki^*^,^ *Crmp1*^−/−^, *Crmp1^ki^*^/^*^ki^*/*SOD1^G93A^*, and *Crmp1*^−/−^/*SOD1^G93A^* mice

To generate *Crmp1*^ki/ki^ mice in which Ser522 was replaced with nonphosphorylatable Ala, we employed CRISPR/Cas9 technology. *Crmp1*^ki/ki^ mice were constructed according to the targeting strategy outlined in Extended Data Figure 2-1*A*. In addition to the Ser522Ala (S522A) substitution, we introduced a *Nar*I digestion site without altering the amino acid sequence to facilitate genotyping. The single guide RNA (sgRNA) targeting mouse *Crmp1* was designed using CHOPCHOP (http://chopchop.cbu.uib.no). The sequence used was as follows: 5′-GTGTTTAGAAGGCGAGGATT-3′. The template DNA for *in vitro* transcription was generated by PCR using a forward primer that consists of the T7 promoter sequence (5′-TTAATACGACTCACTATAGG-3′) followed by the sgRNA sequence and scaffold sequence(5′-GTTTTAGAGCTAGAAATAGCA-3′), and a reverse primer (5′-CACCGACTCGGTGCC-3′). Plasmid DR274 (plasmid #42250; Addgene) was used as the PCR template. The PCR product was purified with the QIAquick Gel Extraction kit (#28706; QIAGEN) and used to synthesize sgRNA using the MEGAshortscript T7 Transcription kit (Thermo Fisher Scientific).

**Figure 1.**
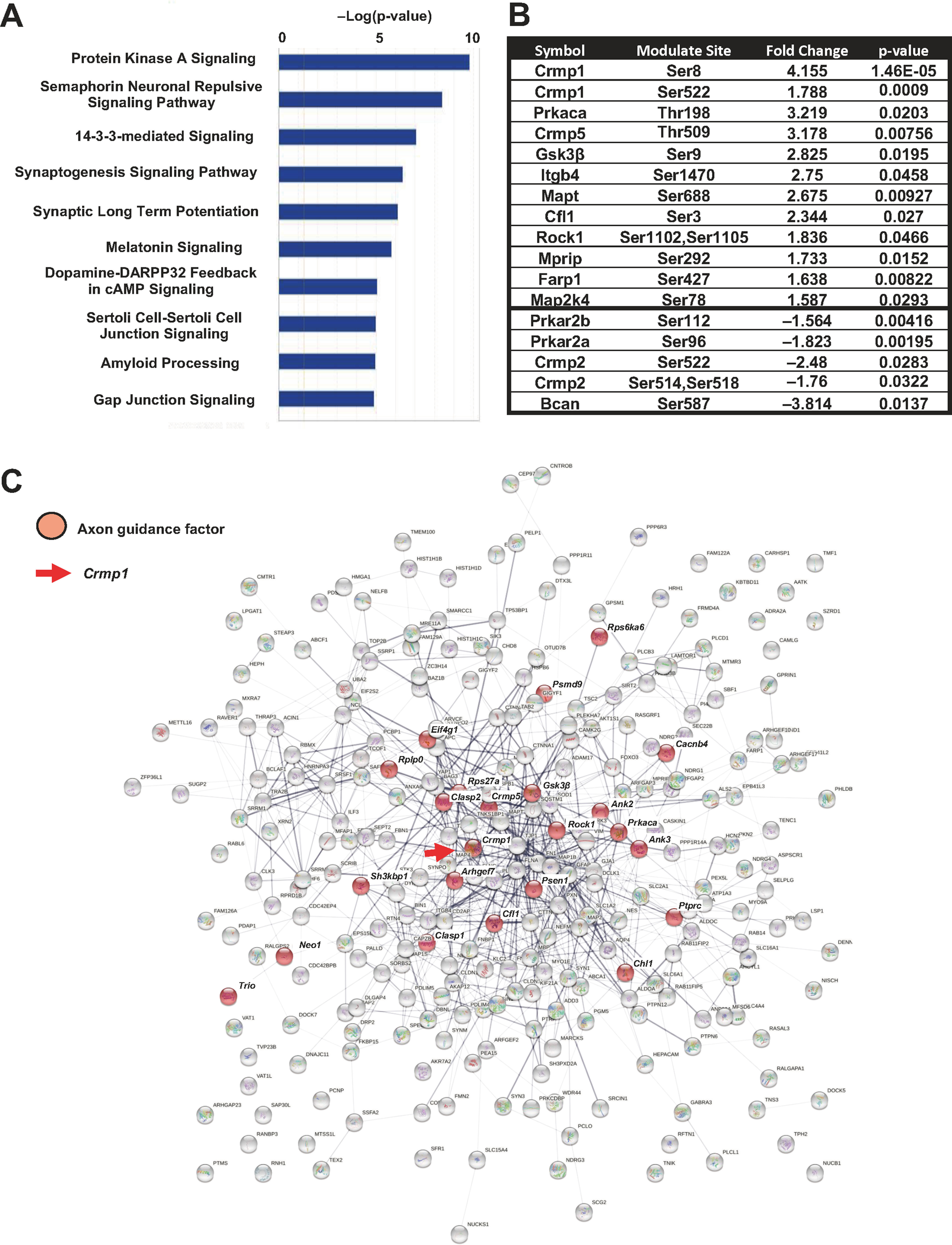
Phosphoproteomic analysis of spinal cords from WT and *SOD1*^G93A^ mice at 20 weeks of age. ***A***, Top 10 canonical pathways identified based on the molecules that were differentially expressed (max fold change >1.5, ANOVA *p *<* *0.05) between WT and *SOD1*^G93A^ mice in phosphoproteomics. ***B***, A list of phosphoproteins in semaphorin neuronal repulsive signaling pathway with significant expression changes in the lumbar spinal cords of *SOD1*^G93A^ mice compared with WT mice (max fold change 1.5; *p *<* *0.05). Phosphorylation sites, fold-change levels, and *p*-value are also shown. ***C***, Protein–protein interaction (PPI) network of differentially upregulated proteins in *SOD1*^G93A^ mice visualized by STRING: https://string-db.org/. Red nodes are phosphoproteins associated with axon guidance in reactome pathway. The red arrow indicates Crmp1.

**Figure 2.**
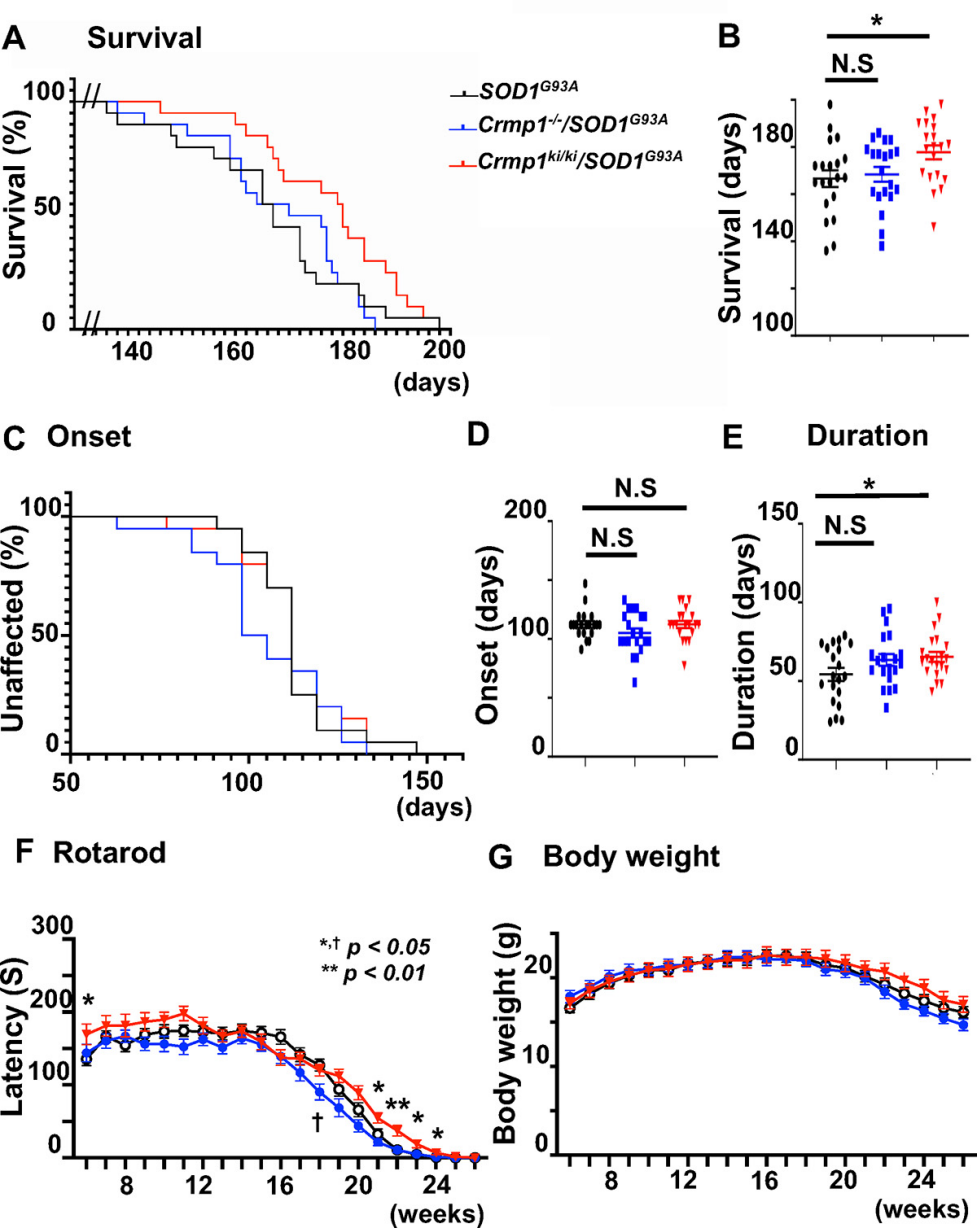
Phenotypic comparisons of *SOD1*^G93A^, *Crmp1*^−/−^/*SOD1*^G93A^, and *Crmp1*^ki/ki^/*SOD1*^G93A^ mice. This figure is supported by Extended Data Figures 2-1, 2-2. ***A***, Kaplan–Meier survival curves for *SOD1*^G93A^ (black), *Crmp1*^−/−^/*SOD1*^G93A^ (blue), and *Crmp1*^ki/ki^/*SOD1*^G93A^ (red) mice. Median survival duration in *SOD1*^G93A^, *Crmp1*^−/−^/*SOD1*^G93A^, and *Crmp1*^ki/ki^/*SOD1*^G93A^ mice was 167, 173, and 180 d, respectively (the log-rank test, *p *=* *0.044). ***B***, The comparative survival durations for three groups (two-way ANOVA, *p <* *0.05). ***C***, Kaplan–Meier curves for disease onset. ***D***, Median disease onset in *SOD1*^G93A^, *Crmp1*^−/−^/*SOD1*^G93A^, and *Crmp1*^ki/ki^/SOD1^G93A^ mice was 112, 102, and 112 d, respectively, and the differences were not significant (the log-rank test, *p *=* *0.797). ***E***, Mean disease duration (days from onset to end stage). Mean disease duration in *SOD1*^G93A^, *Crmp1*^−/−^/*SOD1*^G93A^, and *Crmp1*^ki/ki^/*SOD1*^G93A^ mice was 54, 63, and 65 d, respectively. Moreover, the duration was longer in *Crmp1*^ki/ki^/*SOD1*^G93A^ mice than in *SOD1*^G93A^ mice (one-way ANOVA with Fisher’s LSD test, *p *=* *0.039). ***F***, Rotarod test. *Crmp1*^ki/ki^/*SOD1*^G93A^ mice exhibited a significant improvement in motor function at the late stage (21–24 w) compared with *SOD1*^G93A^ mice, while *Crmp1*^−/−^/*SOD1*^G93A^ showed shorter latency to fall during the rotarod test at 18 w. Values are means ± SD (*SOD1*^G93A^, *n *=* *20; *Crmp1*^−/−^/*SOD1*^G93A^, *n *=* *20; *Crmp1*^ki/ki^/*SOD1*^G93A^, *n *=* *20). *,^†^*p *<* *0.05, **p <* *0.01 (two-way ANOVA with Fisher’s LSD test). ***G***, Body weight. Significant differences were not observed between the three lines of model mice. N.S. = not significant.

10.1523/ENEURO.0133-22.2022.f2-1 Extended Data Figure 2-1 Generation of Crmp1S522A knock-in mice. A, Substituted nucleotides and amino acids are shown as red letters. NarI restriction sites used for genotyping are shown as blue dotted squares. B, Genotyping analysis for mutated alleles by PCR. C, Sanger sequencing electropherograms for the targeted site in WT mice (top), heterozygous (middle), and homozygous knock-in (KI) offspring (bottom). Substitution sites in Crmp1 are indicated by square. D, Western blottings for phosphorylated Crmp1 and Crmp2 protein. Western blottings for WT, Crmp2 homozygous (ki/ki; nonphosphorylatable Crmp2S522A knock-in), Crmp1 heterozygous (ki/+), and homozygous (ki/ki) mouse brain lysates with anti-phospho-Crmp1/2-S522 antibody. Upper bands indicate phospho-Crmp2-S522 (blue arrow) and lower band indicates phospho-Crmp1S522 (red arrow). E, Morphology of E15 DRG growth cones of the WT and Crmp1ki/ki mice embryos treated with Sema3A. In WT DRG explants, Sema3A (1 U/ml) collapsed growth cones. By contrast, growth cone collapse induced by Sema3A was suppressed in Crmp1ki/ki DRG explants. Growth cones were stained with Alexa Fluor 488–phalloidin. Scale bar: 10 μm. F, SemaA concentration-response curve for growth cone collapse of WT and Crmp1ki/ki DRG neurons. Data are mean ± SEM for n = 8; **p < 0.01 by two-way ANOVA with Bonferroni’s multiple comparisons test. G, Accelerated rotarod performance (5–40 rpm/5 min) from 8 to 30 w. No significant differences were detected between WT and Crmp1 KI mice. Download Figure 2-1, TIF file.

sgRNA was purified by phenol-chloroform extraction and ethanol precipitation, and resuspended in OPTI-MEM (Thermo Fisher Scientific). Chemically synthesized single-stranded DNA with the following sequence was used as donor DNA (Nihon Gene Research Laboratories): 5′-GCCAGCTACACCCAAACATGCTGCTCCTGCTCCTTCTGCCAAATCGGCGCCTTCTAAACACCAACCCCCACCCATCCGGAACCTCCACCAGTCC-3′. The mutant sequence to be introduced is underlined. A mixture of sgRNA, Cas9 protein (#632641; Clontech), and donor oligonucleotide was introduced via electroporation into pronuclear stage zygotes generated by IVF. Electroporated embryos were cultured overnight and transferred into the oviducts of 0.5 dpc (days post cotium) pseudopregnant females.

Heterozygous *Crmp1*^ki/+^ males were mated with heterozygous *Crmp1* ^ki/+^ females to generate homozygous *Crmp1*^ki/ki^ mice. Heterozygous, homozygous, and WT alleles were detected by PCR with the following primers: 5′-TGTCTTAGCCTCCCTCCTTT-3′ and 5′-ACCCGCCTAGACTGTGTCTT-3′. PCR cycling conditions were as follows 5 min at 95°C; 1 min at 95°C, 1 min at 57°C, and 2 min at 72°C for 38 cycles, followed by 7 min at 72°C. For PCR, BIOTAQ HS DNA Polymerase (#BIO-21047; Meridian) and Ampdirect^®^ Plus (#P/N 241-08800-98; Shimadzu) were mixed with standard PCR reagents. To distinguish each genotype, PCR products were digested with *Nar*I (#R0191; New England BioLabs) and visualized by electrophoresis on 2% agarose (Extended Data Fig. 2-1*B*). Sanger sequencing was performed to verify successful introduction of either homozygous or heterozygous S522A mutation in *Crmp1* (Extended Data Fig. 2-1*C*). Using brain lysates, we verified suppression of Ser522 phosphorylation in *Crmp1*^ki/ki^ mice (Extended Data Fig. 2-1*D*).

Animals were housed two to five per square plastic cage with wire lids under standard laboratory conditions (23 ± 2°C) on a light/dark cycle (light period, 5 A.M. to 7 P.M.) and free access to food and water. *Crmp1*-deficient (*Crmp1*^−/−^) mice were generated as described previously (Charrier et al., 2006). *SOD1^G93A^* mice were purchased from The Jackson Laboratory (Gurney et al., 1994). We generated *Crmp1*^−/−^/*SOD1^G93A^* and *Crmp1*^ki/ki^/*SOD1*^G93A^ mice by crossing *SOD1*^G93A^ mice with *Crmp1*^−/−^ and *Crmp1^ki^*^/^*^ki^* mice, respectively.

### Behavioral analysis

Body weight measurements and rotarod test were performed weekly. For *Crmp1*^−/−^/*SOD1^G93A^* (*n *=* *20, 10 males and 10 females) and *Crmp1*^ki/ki^/*SOD1^G93A^* mice (*n *=* *20, 10 males and 10 females), behavioral analysis began at six weeks of age (6 w) and continued until 26 w. An accelerating rotarod test was performed, using rotation speeds of 5–40 rpm for 300 s. Mice underwent two trials with an inter-trial interval of >20 min and measurements of the time elapsed to fall from the rotating cylinder were averaged and recorded (Numata-Uematsu et al., 2019). Survival was defined based on the age at which mice could no longer roll over within 30 s after being placed on their back.

### Immunohistochemistry

*SOD1^G93A^*, *Crmp1*^−/−^/*SOD1^G93A^*, *Crmp1*^ki/ki^/*SOD1^G93A^*, and WT mice were anesthetized with 0.3 mg/kg of medetomidine, 4.0 mg/kg of midazolam, and 5.0 mg/kg of butorphanol (M/M/B:0.3/4/5) and killed at 20 w (Kawai et al., 2011). Mice were perfused with PBS followed by 4% paraformaldehyde (PFA) in PBS. Lumbar spinal cords were dissected and tissues were immediately fixed in 4% PFA and embedded in paraffin. Blocked spinal cords were cut into 6-μm cross-sections that were later stained with ChAT (1:100, #AB144P, RRID:AB_2079751; Millipore) for quantitative analysis of motor neurons (WT: *n *=* *4, all females; *SOD1*^G93A^: *n *=* *7, 5 males and 2 females; *Crmp1*^−/−^/*SOD1*^G93A^: *n *=* *8, 5 males and 3 females; *Crmp1*^ki/ki^/*SOD1*^G93A^: *n *=* *7, 3 males and 4 females) and glial cell evaluations (WT: *n *=* *5, 1 male and 4 females; *SOD1*^G93A^: *n *=* *5, 3 males and 2 females; *Crmp1*^−/−^/*SOD1*^G93A^: *n *=* *5, 3 males and 2 females; *Crmp1*^ki/ki^/*SOD1*^G93A^: *n *=* *5, 2 males and 3 females).

For immunofluorescence staining, paraffin-embedded sections were permeabilized with 0.1% Triton X-100 in PBS for 20 min, blocked with 5% bovine serum for 30 min, and incubated overnight with rat anti-rabbit Iba1 polyclonal antibody (1:400, #019-19741, RRID:AB_839504; FUJIFILM Wako), anti-mouse glial fibrillary acidic protein (GFAP) monoclonal antibody (1:400, #G3893, RRID:AB_477010; Sigma-Aldrich), anti-mouse neuronal nuclei (NeuN) monoclonal antibody (1:400, #MAB377, RRID:AB_2298772; Millipore), and anti-rabbit phospho-Ser522 Crmp1/2 (1:400; FUJIFILM Wako; Uchida et al., 2005). Sections were subsequently incubated with secondary antibody conjugated with either Alexa Fluor 488 (1:1000, #A-11034, RRID:AB_2576217; Thermo Fisher Scientific) or Alexa Fluor 568 (1:1000, #A-11019, RRID:AB_143162; Thermo Fisher Scientific). Immunostained cells were analyzed in four random fields/sections using a deconvolution fluorescence microscope system (BZ-X800; Keyence).

### Assessment of NMJ innervation

Tibialis anterior (TA) muscles were dissected and fixed with 2% PFA in PBS for 20 min at room temperature. Fixation was followed by cryoprotection with 20% sucrose in PBS overnight. TA muscles were frozen in Tissue-Tek^®^ O.C.T. Compound (Sakura Finetek), and 40-μm-thick sections were made using a cryostat (Tissue-Tek^®^ Cryo3^®^; Sakura Finetek). Three sections were collected per animal on glass slides. Muscle sections were stained with mouse anti-synaptic vesicle protein (1:100, #SV2, RRID:AB_2315387; DSHB) and mouse anti-NFs (1:2000, #2H3, RRID:AB_531793; DSHB) overnight at 4°C. Alexa Fluor 594-labeled α-bungarotoxin (α-BTX; 1:500, #B-13423; Thermo Fisher Scientific) and mouse Alexa Fluor 488 (1:500, #A-11017, RRID:AB_2534084; Thermo Fisher Scientific) were subsequently added to the samples, followed by overnight incubation at room temperature. Images were obtained with a deconvolution fluorescence microscope system (BZ-X800, BZ-X9000; Keyence). Colocalization of NF and α-BTX was verified by creating z-stack images at 40× magnification.

The percentage of neuromuscular innervation was measured at 42-96 randomly selected synaptic sites per mouse (*n *=* *3 in each group, all males). Endplate occupancy was determined by assessing the extent of overlap of axon terminal signal (labeled by SV2/2H3) with endplate signal (labeled by α-BTX). The degree of denervation was determined as previously described (Numata-Uematsu et al., 2019). Endplates were scored as “denervated” when <5% of the endplate was deemed occupied by the axon terminal; “fully innervated” means >95% occupancy; “partially innervated” means intermediate occupancy.

### Western blotting

Brain (*n *=* *1 in each group, all males) and spinal cord samples (*n *=* *4 in each group, all males) were homogenized in lysis buffer (20 mm Tris-HCl, pH 8.0, 150 mm NaCl, 1% Nonidet P-40) supplemented with one tablet of cOmplete, Mini1% phosphatase inhibitor cocktail 2 (# P5726; Sigma-Aldrich) and 1% phosphatase inhibitor cocktail 3 (#P0044; Sigma-Aldrich). Lysates were centrifuged at 10,000 × *g* for 20 min at 4°C, and supernatants were normalized to total protein concentration. The samples were used for Western blotting with rabbit anti-phosphoS522 Crmp1/2 (1:5000; FUJIFILM Wako; Uchida et al., 2005), rabbit anti-Crmp1 (1:5000, #ab199722; Abcam), and mouse anti-β-actin (1:10,000, #3700, RRID:AB_2242334; Cell Signaling Technology) antibodies.

### Dorsal root ganglion (DRG) culture and growth cone collapse assay

A primary culture of mouse DRG neurons was prepared as previously described (Kawashima et al., 2021). Briefly, DRGs were dissected from embryonic day 14–15 WT (C57B6/J) and Crmp1^ki/ki^ mice and plated on glass-bottom culture dishes precoated sequentially with poly-L-lysine (100 μg/ml; #P4832; Sigma-Aldrich) and mouse laminin (8 μg/ml; #354232; Corning). DRG explants were subsequently cultured in 250 μl/well of Neurobasal medium (#21103049; Thermo Fisher Scientific) supplemented with 2% B-27 (#17504044; Thermo Fisher Scientific), 1 mm GlutaMax (#35050061; Thermo Fisher Scientific), 20 ng/ml NGF, 50 U/ml penicillin, and 50 μg/ml streptomycin for 1 d at 37°C. The explants were stimulated with either 0.5, 1, or 3 U/ml purified recombinant chick Sema3A for 10 min at 37°C and fixed with PBS containing 2% formaldehyde and 10% sucrose. Growth cones were stained with Alexa Fluor 488 phalloidin (#A12379; Thermo Fisher Scientific). The growth cones without lamellipodia or filopodia were scored as collapsed. In each condition, >50 growth cones were examined. Sema3A at 1 U/ml concentration induced 50% collapsed growth cones of chick E7 DRG neurons (Nakamura et al., 2014). Growth cone images were captured with a BZ-X800 microscope at 40× magnification (Keyence).

### Proteomics

Lumbar spinal cords were isolated from 20-week-old mice that were first sedated by anesthesia with M/M/B:0.3/4/5 and killed thereafter by rapid decapitation. Dissected spinal cords were stored at −80°C until use. Lumbar spinal cords were sonicated on ice at 20 kHz for 30 s a total of four times in lysis buffer [50 mm NH_4_HCO_3_, 8 m urea, 4% sodium deoxycholate, 1% phosphatase inhibitor cocktail 2 (#P5726; Sigma-Aldrich), 1% phosphatase inhibitor cocktail 3 (#P0044; Sigma-Aldrich), 1% protease inhibitor mix (#03969-21; Nacalai Tesque)] using a Branson cell disruptor. Cleared spinal lysate was obtained by centrifugation at 15,000 × *g* for 15 min at 4°C. Proteins were precipitated with four volumes of cold acetone and resuspended in 200 μl of lysis buffer. A total of 600-μg protein extracted from each spinal sample was reduced with 10 mm dithiothreitol and alkylated with 12.5 mm iodoacetamide. Proteins were diluted with three volumes of 50 mm NH_4_HCO_3_ before digestion with trypsin (Promega) at an enzyme:substrate ratio of 1:20 overnight at 37°C. Sodium deoxycholate was removed from samples using the phase-transfer surfactant method using ethyl acetate (Masuda et al., 2008). After desalting using an OASISHLB 1 ml (Waters), phosphopeptide enrichment was performed with a homemade TiO_2_-C8 tip column using solution compositions specified by the Titansphere Phos-TiO kit (GL Sciences). Our homemade TiO_2_-C8 tip-column was made from a 200-μl pipette tip (D200; Gilson) by layering 3 mg of TiO_2_ particles (GL Sciences) on top of C8 disk filters (Empore C8; 3M Corporation). After drying, the peptides obtained were dissolved in 0.1% formic acid and 2% acetonitrile (ACN) and analyzed using a Q-Exactive mass spectrometer (Thermo Fisher Scientific) equipped with an UltiMate 3000 LC system (Thermo Fisher Scientific). Peptides were loaded on a trap column (100 μm × 20 mm, C18, 5 μm, 100 Å; Thermo Fisher Scientific) and subsequently separated on a Nano HPLC capillary column (75 μm × 180 mm, C18, 3 μm; Nikkyo Technos). Buffer A was 0.1% formic acid in 2% ACN while buffer B was 0.1% formic acid in 95% ACN. Peptides were eluted with a linear gradient of 2–33% buffer B for 120 min.

Label-free quantitative analysis was conducted using the software Progenesis QI for proteomics (Nonlinear Dynamics). For protein and peptide identification, peak lists were created using the software Progenesis QI for proteomics and searched against mouse protein sequences in the UniProtKB/Swiss-Prot database http://www.uniprot.org/ using the MASCOT software (Matrix Science). Basic search parameters were as follows: trypsin digestion with two missed cleavages permitted; peptide mass tolerance, ±5 ppm; fragment mass tolerance, ±0.05 Da; usual variable modifications, methionine oxidation, cysteine carbamidomethylation, protein N-terminal acetylation, and N-terminal carbamylation. For phosphoproteomic analysis, additional variable modification parameters for analyzing MS data were phosphorylation of serine, threonine, and tyrosine. Identifications were filtered at a 1% false discovery rate and significance peptide score ≥30. Protein interaction analysis was conducted with the online tool STRING (https://string-db.org, default settings; PMID 15608232). Ingenuity pathway analysis (IPA; content version: 60467501/62089861, release date: 2020-11-19/2021-02-17; QIAGEN) was used for pathway analysis. Females and males were used for phosphoproteomics and proteomics, respectively.

### Pathway analysis

Genes mapped from significantly upregulated or downregulated peptides and phosphorylated peptides were used to identify cellular and molecular processes, pathways and clusters using STRING and IPA software. Activation *z* scores were calculated using IPA’s *z* score algorithm to predict the overall activation or inhibition of the functional cellular processes/pathways and upstream regulators identified. A positive *z* score implies an overall predicted activation of the process/pathway/upstream regulator, whereas a negative *z* score implies an overall predicted inhibition or downregulation of the pathway/process/upstream regulator. IPA considers *z* scores of ≥2 indicative of significant activation while *z* scores ≤−2 are indicative of inhibition. Cellular processes/upstream regulators with no *z* scores imply that IPA cannot generate prediction states for these functionalities.

### Statistical analysis

Statistical evaluation of behavioral analysis, immunohistochemistry, and Western blotting results was performed using Prism 8 (GraphPad Software). For Rotarod test and body weight data, a two-way ANOVA with Fisher’s LSD test was used at each age. The Kaplan–Meier method was used to analyze survival and onset in each *SOD1^G93A^* mouse strain. Immunohistochemistry and Western blotting results were analyzed using either ImageJ or ImageQuant software (GE Healthcare).

### Data availability

All mass spectrometry proteomics data have been deposited with the ProteomeXchange Consortium (http://www.proteomexchange.org) via the jPOST (https://jpostdb.org) partner repository with the dataset identifier PXD030651 (https://repository.jpostdb.org/preview/13182122262802fe459f41, access key: 5727). All data are fully available without restriction.

## Results

### Phosphoproteomic analysis of the spinal cord of *SOD1*^G93A^ mice at 20 weeks of age

To characterize posttranslational changes associated with ALS, we determined the phosphoproteomic profile of the spinal cord of *SOD1^G93A^* (*n *=* *5) mice and compared it with that of WT (*n *=* *4) mice at 20 weeks of age. Canonical pathway analysis using IPA identified semaphorin neuronal repulsive signaling pathway as one of the major pathways affected in the *SOD1^G93A^* mouse spinal cord (Fig. 1*A*). Thirteen upregulated and six downregulated phosphorylation sites were specified in 11 and 4 proteins that make up this pathway, respectively (Fig. 1*B*). Signaling proteins downstream of Sema3A included Crmp1, Crmp2, Crmp5, Gsk3β, and Farp1. For Crmp1 in particular, phosphorylation was significantly higher at both Ser8 and Ser522 (Fig. 1*B*). The interactome of upregulated phosphopeptides (fold change >1.5, *p* < 0.05) in *SOD1*^G93A^ mice was visualized by STRING: functional protein association networks (https://string-db.org/; Fig. 1*C*). We again found changes in phosphoproteins associated with axon guidance, including Crmp1, Crmp5, GSK3β, Cfl1, and Rock1. Sema3A signaling has a well-established relationship with Crmp1 phosphorylation at Ser522, but not at Ser8. Therefore, in this study, we focused on the role of CRMP1 Ser522 phosphorylation in ALS pathogenesis.

### The effects of total depletion of Crmp1 and of blocking Crmp1 phosphorylation at Ser522 on phenotypes of ALS mice

To determine the roles of total CRMP1 and CRMP1 phosphorylation at Ser522 in ALS pathogenesis, we employed *Crmp1* knock-out (*Crmp1*^−/−^) mice (Cole et al., 2006) and *Crmp1* knock-in (*Crmp1^ki/ki^*) mice, the latter of which we newly established by introducing the S522A mutation to block Crmp1 phosphorylation at Ser522 using the CRISPR/Cas9 system (Extended Data Fig. 2-1*A*). Sanger sequencing confirmed successful introduction of the mutation (Extended Data Fig. 2-1*B*,*C*), and Western blotting failed to detect Crmp1-Ser522 phosphorylation in *Crmp1^ki^*^/^*^ki^* mice (Extended Data Fig. 2-1*D*). Increased Crmp2-Ser522 phosphorylation in *Crmp1^ki^*^/^*^+^* and *Crmp1^ki^*^/^*^ki^* mice compared with WT mice might be attributed to the compensatory effect of Crmp2.

Next, we analyzed Sema3A response in cultured DRG neurons from *Crmp1^ki/ki^* mice. The Sema3A-induced growth cone collapse response of *Crmp1^ki/ki^* DRG neurons at E14–15 was significantly lower than that of WT neurons measured at 1 and 3 U/ml Sema3A (*F*_(1,56)_ = 20.67, 0.5 U/ml: *p *=* *0.067, 1 U/ml: *p *=* *0.008, 3 U/ml: *p *=* *0.006, two-way repeated measures ANOVA followed by Bonferroni’s multiple-comparisons test; Extended Data Fig. 2-1*E*,*F*). This result indicates that blocking Crmp1 phosphorylation at Ser522 attenuates the Sema3A signal in mouse DRG neurons. Furthermore, we evaluated the motor function of *Crmp1^ki/ki^* mice and no significant differences were observed between WT and *Crmp1^ki/ki^* mice in a rotarod test (Extended Data Fig. 2-1*G*). *Crmp1*^−/−^ mice have been shown to have normal motor function (Yamashita et al., 2013).

These *Crmp1* mutant mice were subsequently crossed with *SOD1^G93A^* mice and resultant *Crmp1*^−/−^/*SOD1^G93A^* and *Crmp1^ki^*^/^*^ki^*/*SOD1^G93A^* mice were compared with *SOD1^G93A^* mice. We used Western blotting to measure the levels of phospho-Crmp1-Ser522 and total Crmp1 in the spinal cord of these model mice at 20 weeks of age. The levels of phospho-Crmp1-Ser522 normalized to total Crmp1 were higher in *SOD1^G93A^* mice (*t*_(6)_ = 8.797, *p *=* *0.0001, unpaired *t* test) than in WT mice (Extended Data Fig. 2-2*A*,*B*), in agreement with our phosphoproteomics results, and they were completely suppressed in *Crmp1* mutant mice. By contrast, total Crmp1 was reduced in *SOD1^G93A^* mice (*F*_(2,9)_ = 0.092, *p *=* *0.042, one-way ANOVA with Uncorrected Fisher’s LSD), absent from *Crmp1*^−/−^/*SOD1^G93A^* mice, but at WT levels in *Crmp1^ki^*^/^*^ki^*/*SOD1^G93A^* mice (Extended Data Fig. 2-2*A*,*C*). To investigate the types of cells expressing phospho-Crmp1, we performed immunofluorescence analysis in the ventral horn of the lumbar spinal cord of *SOD1*^G93A^ mice at 20 weeks of age using anti–phospho-Ser522 Crmp1/2 antibody. Phospho-Crmp1/2-Ser522 was colocalized with NeuN (neuron; Extended Data Fig. 2-2*D*) but not with GFAP (astrocyte; Extended Data Fig. 2-2*E*).

10.1523/ENEURO.0133-22.2022.f2-2 Extended Data Figure 2-2 Relative levels of phosphor-Crmp1-S522 and total Crmp1 in congenic SOD1G93A mouse strains. A, Western blottings for phospho-Crmp1-S522 and total Crmp1 protein level in the spinal cord of WT and SOD1G93A mutant mouse strains at 20 w. Upper bands correspond to phospho-Crmp2-S522 (blue arrow) while lower bands correspond to phospho-Crmp1-S522 (red arrow). B, The levels of phospho-Crmp1-S522 normalized to total Crmp1 are significantly higher in SOD1G93A mice than WT mice. ***p < 0.001 by unpaired t test. Statistical significance was determined as follows: WT versus SOD1G93A; p = 0.0001 by unpaired t test. C, Total Crmp1 protein was decreased in SOD1G93A mice compared with WT mice. *p < 0.05 by one-way ANOVA with uncorrected Fisher’s LSD. Statistical significance was determined as follows: WT versus SOD1G93A; p = 0.042, WT versus Crmp1ki/ki/SOD1G93A; p = 0.0549. D, Immunofluorescence data show that phospho-Crmp1/2-S522 (red) is co-localized with NeuN (green), as shown in the merged image (yellow) of the ventral horn of the lumbar spinal cord from SOD1G93A mice at 20 w. Nuclei were counterstained by Hoechst (blue). Scale bar: 50 μm. E, Phospho-Crmp1/2 S522 (red) and GFAP (green) were not colocalized in the ventral horn of lumbar spinal cord from SOD1G93A mice at 20 w. Nuclei were counterstained by Hoechst (blue). Scale bar: 50 μm. Download Figure 2-2, TIF file.

Phenotypic analysis revealed that selective inhibition of Crmp1 phosphorylation at Ser522 in *Crmp1^ki^*^/^*^ki^*/*SOD1^G93A^* mice, but not the complete knock-out of *Crmp1* in *Crmp1*^−/−^/*SOD1^G93A^* mice, prolonged survival duration relative to *SOD1^G93A^* mice (median survival: *SOD1^G93A^*, 167 d; *Crmp1*^−/−^/*SOD1^G93A^*, 173 d; *Crmp1^ki^*^/^*^ki^*/*SOD1^G93A^* 180 d, *p *=* *0.044 with log-rank test; Fig. 2*A*,*B*). In detail, the disease duration in *SOD1^G93A^*, *Crmp1*^−/−^/*SOD1^G93A^*, and *Crmp1^ki^*^/^*^ki^*/*SOD1^G93A^* mice was 54, 63, and 65 d, respectively. Moreover, the disease duration was longer in *Crmp1^ki^*^/^*^ki^*/*SOD1^G93A^* mice than in *SOD1^G93A^* mice (*p *=* *0.039), but the disease onset in all three types of mice remained unchanged (112, 102, and 112 d, respectively; Fig. 2*C–E*). *Crmp1^ki^*^/^*^ki^*/*SOD1^G93A^* and *Crmp1*^−/−^/*SOD1^G93A^* mice showed longer (6 w; *p *=* *0.049, 21 w; *p *=* *0.032, 22 w; *p *=* *0.004, 23 w; *p *=* *0.019, 24 w; *p *=* *0.025 by two-way ANOVA with Fisher’s LSD test) and shorter (18 w; *p *=* *0.011) latency to fall in rotarod test, respectively, compared with *SOD1^G93A^* mice (Fig. 2*F*). Genetic modifications of *Crmp1* in *SOD1^G93A^* mice did not affect body weight (Fig. 2*G*). These results indicate that abolishing Crmp1 phosphorylation at Ser522, without depleting Crmp1 altogether, may ameliorate the phenotypes of *SOD1^G93A^* mice.

### Pathologic evaluations of *Crmp1*^−/−^/*SOD1^G93A^* and *Crmp1^ki^*^/^*^ki^*/*SOD1^G93A^* mice

**Figure 3.**
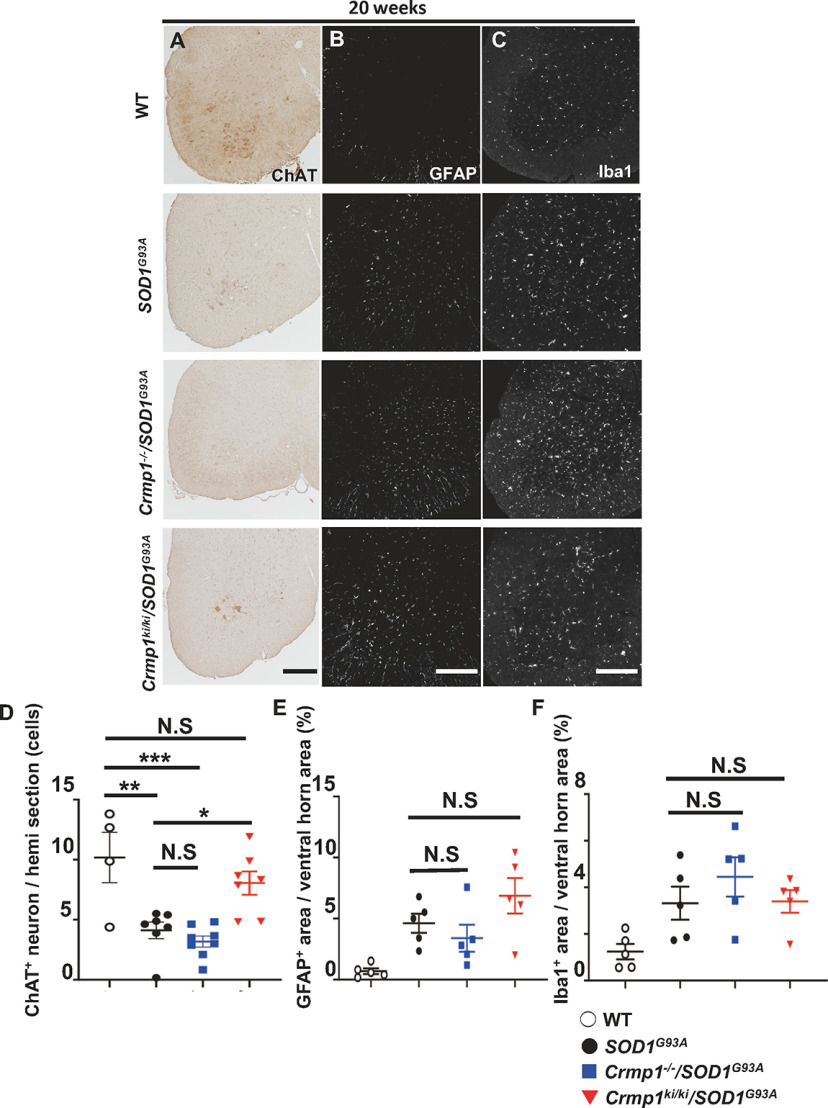
Motor neuron degeneration and gliosis. This figure is supported by Extended Data Figure 3-1. ***A***, Representative images of ChAT-stained motor neurons in the lumbar spinal cord of mice at 20 w. GFAP-immunostained (***B***) and Iba1-immunostained (***C***) images of the lumbar spinal cord. Scale bar: 200 μm. ***D***, Counts of ChAT-positive neurons in hemi sections of the lumbar spinal cord (WT; *n *=* *4, *SOD1*^G93A^; *n *=* *7, *Crmp1*^−/−^/*SOD1*^G93A^; *n *= 8, *Crmp1*^ki/ki^/*SOD1*^G93A^; *n *=* *7). Significance was determined by one-way ANOVA with Tukey’s multiple comparisons test as follows: WT versus *SOD1*^G93A^; *p *=* *0.003, WT versus *Crmp1*^−/−^/*SOD1*^G93A^; *p *<* *0.001, *SOD1*^G93A^ versus *Crmp1*^−/−^/*SOD1*^G93A^; *p *=* *0.871, WT versus *Crmp1*^ki/ki^/SOD1^G93A^; *p *=* *0.496, *SOD1*^G93A^ versus *Crmp1*^ki/ki^/SOD1^G93A^; *p *=* *0.0253. Percentage of GFAP-positive (***E***) and Iba1-positive (***F***) area within the ventral horn area (WT; *n *=* *5, *SOD1*^G93A^; *n *=* *5, *Crmp1*^−/−^/*SOD1*^G93A^; *n *=* *5, *Crmp1*^ki/ki^/*SOD1*^G93A^; *n *=* *5). Significance was determined by one-way ANOVA with Dunnett’s multiple comparisons test as follows: (***E***) *SOD1*^G93A^ versus *Crmp1*^−/−^/*SOD1*^G93A^; *p *=* *0.723, *SOD1*^G93A^ versus *Crmp1*^ki/ki^/*SOD1*^G93A^; *p *=* *0.292, (***F***) *SOD1*^G93A^ versus *Crmp1*^−/−^/*SOD1*^G93A^; *p *=* *0.457, *SOD1*^G93A^ versus *Crmp1*^ki/ki^/SOD1^G93A^; *p *=* *0.999. Values are means ± SEM; *p <* *0.05, **p <* *0.01, ***p <* *0.001, N.S. = not significant as determined by one-way ANOVA.

To examine the effect of Crmp1 depletion and selective inhibition of Crmp1 phosphorylation at Ser522 on mutant SOD1–induced neurodegeneration in mice, we measured the number of residual motor neurons in the anterior horn of the lumbar spinal cord from WT (*n *=* *4), *SOD1*^G93A^ (*n *=* *7), *Crmp1*^−/−^/*SOD1*^G93A^ (*n *=* *8), and *Crmp1^ki^*^/^*^ki^*/*SOD1^G93A^* (*n *=* *7) mice at 20 weeks of age. The loss of motor neurons in *Crmp1*^−/−^/*SOD1^G93A^* mice was likely to be more evident than in *SOD1^G93A^* mice, but the difference was not statistically significant (*p *=* *0.871 by one-way ANOVA with Tukey’s; Fig. 3*A*,*D*). Compatible with our phenotypic findings, the numbers of motor neurons were significantly preserved in *Crmp1^ki^*^/^*^ki^*/*SOD1^G93A^* mice (WT vs *Crmp1^ki^*^/^*^ki^*/*SOD1^G93A^*; *p *=* *0.496, *SOD1^G93A^* vs *Crmp1^ki^*^/^*^ki^*/*SOD1^G93A^*; *p *=* *0.0253 by one-way ANOVA with Tukey’s; Fig. 3*A*,*D*). However, proliferation of microglia and astroglia showed no significant differences in every *SOD1*^G93A^ mouse strain used in this study (Fig. 3*B*,*C*,*E*,*F*).

10.1523/ENEURO.0133-22.2022.f3-1 Extended Data Figure 3-1 Proteomic analysis and Western blotting of the spinal cord from SOD1G93A, Crmp1–/–/SOD1G93A, and Crmp1ki/ki/SOD1G93A mice. A, Top 10 canonical pathways identified by proteomics analysis of molecules differentially expressed (max fold change >1.5, ANOVA p < 0.05) between SOD1G93A and Crmp1–/–/SOD1G93A mice. B, Top 10 canonical pathways identified by proteomics analysis of molecules differentially expressed (max fold change >1.5, ANOVA with p < 0.05) between SOD1G93A and Crmp1ki/ki/SOD1G93A mice. C, Clustering analysis of differentially expressed canonical pathways between Crmp1–/–/SOD1G93A and Crmp1ki/ki/SOD1G93A mice. Download Figure 3-1, TIF file.

**Figure 4.**
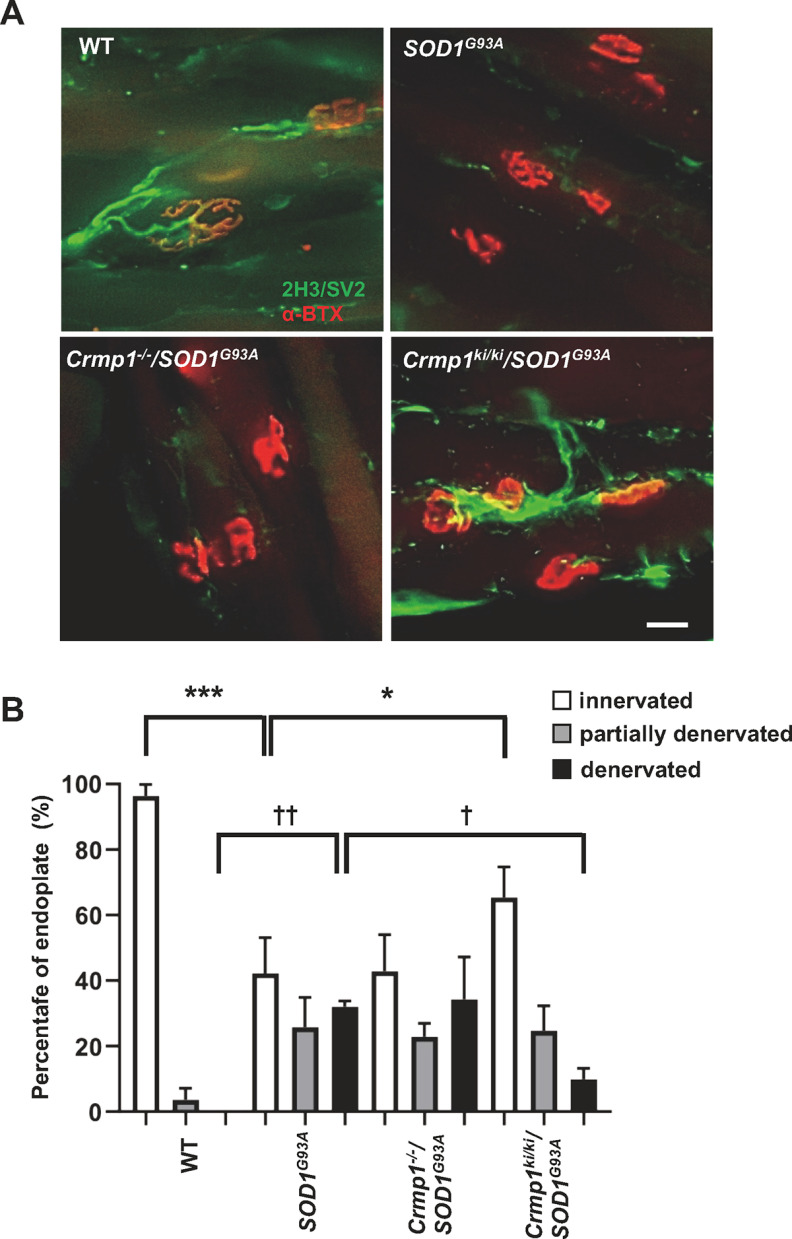
Blocking Crmp1 phosphorylation at S522 delays denervation at NMJs. ***A***, Representative photomicrographs of NMJs in fixed TA muscles. Nerve axons (green) are stained with 2H3 (NF) plus SV2 (synaptic vesicles) and postsynaptic acetylcholine receptors (red) are stained with α-BTX. Scale bar: 20 μm. ***B***, Crmp1 S522A expression increases endplate occupancy in TA muscle of *SOD1*^G93A^ mice. The figure shows the percentage of fully innervated (fully occupied), partially denervated (partially occupied), and denervated endplates of the axon terminals in TA muscles from mice with the indicated genotypes. Statistical significance was determined as follows: innervated: WT versus *SOD1^G93A^*; *p *<* *0.001, *SOD1^G93A^* versus *Crmp1*^−/−^/*SOD1^G93A^*; *p *=* *0.999, *SOD1^G93A^* versus *Crmp1^ki^*^/^*^ki^*/*SOD1^G93A^*; *p *=* *0.038, denervated: WT versus *SOD1^G93A^*; *p *<* *0.001, *SOD1^G93A^* versus *Crmp1*^−/−^/*SOD1^G93A^*; *p *=* *0.955, *SOD1^G93A^* versus *Crmp1^ki^*^/^*^ki^*/*SOD1^G93A^*; *p *=* *0.01, by one-way ANOVA with Dunnett’s multiple comparisons test. Values are means ± SEM (*n *=* *3); *,^†^*p *<* *0.05, ^††^*p *<* *0.01, ***p <* *0.001 by one-way ANOVA with Dunnett’s multiple comparisons test.

We also evaluated the NMJ in the TA muscle of *SOD1^G93A^*, *Crmp1*^−/−^/*SOD1^G93A^*, and *Crmp1^ki^*^/^*^ki^*/*SOD1^G93A^* mice. Compared with NMJs of *SOD1*^G93A^ mice (innervated: 42.2 ± 6.3%, denervated: 32.0 ± 1.0%), *Crmp1^ki^*^/^*^ki^*/*SOD1^G93A^* mice showed more innervated (65.4 ± 5.4%, *p *=* *0.038, by one-way ANOVA with Dunnett’s multiple comparisons test) and fewer denervated NMJs (9.9 ± 1.9%, *p *=* *0.01, by one-way ANOVA with Dunnett’s multiple comparisons test) at 140 d, while *Crmp1*^−/−^/*SOD1^G93A^* mice showed no significant difference (innervated: 42.9 ± 6.4%, denervated: 34.3 ± 7.5%; Fig. 4*A*,*B*). These results further support the notion that inhibiting phosphorylation of Crmp1 at Ser522 can ameliorate mutant SOD1-induced neurodegeneration in mice.

### Total depletion of Crmp1 and blocking Crmp1 phosphorylation at Ser522 differentially regulated the sirtuin signaling pathway in *SOD1^G93A^* mice

Finally, we investigated the molecular basis for differential clinical and pathologic phenotypes observed between *Crmp1*^−/−^/*SOD1^G93A^* and *Crmp1^ki^*^/^*^ki^*/*SOD1^G93A^* mice. For this purpose, we performed the proteomic analysis of the lumbar spinal cord of *Crmp1*^−/−^/*SOD1^G93A^* and *Crmp1^ki^*^/^*^ki^*/*SOD1^G93A^* mice in comparison with *SOD1^G93A^* mice. Using IPA, we visualized the canonical pathways affected in each *SOD1^G93A^* strain. The top 10 canonical pathways affected in *Crmp1*^−/−^/*SOD1^G93A^* and *Crmp1^ki^*^/^*^ki^*/*SOD1^G93A^* mice are shown in Extended Data Figure 3-1*A*,*B*, respectively. Extended Data Figure 3-1*C* shows the results of clustering analysis for SOD1^G93A^ versus *Crmp1*^−/−^/*SOD1^G93A^* mice and *SOD1^G93A^* versus *Crmp1^ki^*^/^*^ki^*/*SOD1^G93A^* mice. Intriguingly, the sirtuin signaling pathway was differently affected in *Crmp1*^−/−^/*SOD1^G93A^* (downregulated) and *Crmp1^ki^*^/^*^ki^*/*SOD1^G93A^* mice (upregulated).

## Discussion

The pathogenesis of ALS involves diverse pathways, including oxidative damage, disruption of protein clearance, mitochondrial dysfunction, apoptosis, axonal transport defects, growth factor deficiency, glial cell pathology, glutamate excitotoxicity, and disruptions in RNA metabolism (Rothstein, 2009; Taylor et al., 2016).

In this study, we performed phosphoproteomic analysis to comprehensively characterize specifically phosphorylated proteins in *SOD1^G93A^* ALS model mice and identified semaphorin neuronal repulsive signaling pathway as one of the major affected pathways (Fig. 1). Sema3A expression was previously shown to be elevated in the motor cortex of ALS patients, although results were less distinct in the spinal cord (Körner et al., 2016). Higher levels of Sema3A in the terminal Schwann cells of *SOD1^G93A^* mice suppressed nerve terminal plasticity and induced motor neuron death (De Winter et al., 2006). These past findings strongly suggest that Sema3A signaling is involved in ALS pathogenesis. Moreover, our phosphoproteomics analysis of the spinal cord of *SOD1^G93A^* mice detected enhanced phosphorylation of Crmp1, Crmp5, GSK3β, and Farp1, all downstream proteins involved in Sema3A signaling (Fig. 1*B*). In particular, we focused on the enhanced phosphorylation of Crmp1 Ser522, which was confirmed by the significantly elevated ratio of phospho-Crmp1-Ser522 to total Crmp1 in *SOD1^G93A^* mice compared with WT mice in Western blotting (Extended Data Fig. 2-2*B*).

Because phospho-antibody against Ser522 has an identical phosphorylation consensus motif for both Crmp1 and Crmp2, it can discriminate these two Crmps by Western blotting (Extended Data Fig. 2-2*A*) but not by immunohistochemistry. Despite this limitation, we characterized the types of cells with Crmp1 phosphorylation using immunofluorescence analysis involving anti–phospho-Ser522 Crmp1/2 antibody. As shown in Extended Data Figure 2-2*D*,*E*, Crmp1/2 in the ventral horn of the lumbar spinal cord was localized in neurons but not in astrocytes, which indicates that elevated phosphorylation of Crmp1 Ser522 may largely occur in neurons. This is also compatible with the previous finding that glial cells do not express CRMP1 protein (Bretin et al., 2005; Luo et al., 2012).

Elevated Crmp1 phosphorylation in the spinal cord of *SOD1^G93A^* mice may be explained by the fact that Sema3A signaling hyperactivates the complex of Cdk5 and p25, an activator of Cdk5, in the brain and spinal cord in a mouse model of ALS as well as in ALS patients (Nguyen et al., 2001; Klinman and Holzbaur, 2015; Bk et al., 2019). Excessive Cdk5 activity is associated with induction of neuronal loss (Cheung and Ip, 2012) and Cdk5 inhibition in the motor neurons prevents motor neuronal death in ALS model mice (Bk et al., 2019). Moreover, primary cultured DRG neurons from *Crmp1^ki^*^/^*^ki^* mice expressing the *Crmp1*^S522A^ mutant were less sensitive to Sema3A stimulation than those from WT mice (Extended Data Fig. 2-1*E*,*F*), in contrast to a previous finding that ectopic expression of the Crmp1^S522D^ phosphomimetic mutant in DRG neurons potentiated the Sema3A-induced growth cone collapse response (Nakamura et al., 2014).

Therefore, we hypothesized that inhibition of Crmp1 phosphorylation at Ser522 ameliorates disease progression in *SOD1^G93A^* mice. In fact, motor function and survival were improved in *Crmp1*^ki/ki^/*SOD1*^G93A^ mice (Fig. 2*A*,*B*,*F*). Reflecting these phenotypic improvements, the residual motor neurons and innervation of NMJs were significantly preserved in *Crmp1^ki^*^/^*^ki^*/*SOD1^G93A^* mice (Figs. 3*A*,*D*, 4*A*,*B*). By contrast, microgliosis and astrocytosis were not affected by *Crmp1* modification in *SOD1*^G93A^ mice (Fig. 3*B*,*C*,*E*,*F*), which is consistent with the previous finding that depletion of *Epha4* in *SOD1*^G93A^ mice attenuates motor neuron degeneration without altering gliosis (Van Hoecke et al., 2012). Because both Crmp1 and Epha4 are axon guidance proteins and are expressed only in neurons (Bretin et al., 2005; Luo et al., 2012; Van Hoecke et al., 2012), their alteration in *SOD1*^G93A^ mice may affect neurons but not glial cells. Our results indicate that blocking Ser522 phosphorylation in Crmp1 has a protective effect on neuronal pathology of ALS. Previously reported adverse effects of Cdk5 activity in ALS models (Nguyen et al., 2001; Klinman and Holzbaur, 2015) may be mediated in part by Crmp1 phosphorylation.

In addition to the differential Crmp1 phosphorylation between WT and *SOD1*^G93A^ mice, expression of total Crmp1 was significantly reduced in the spinal cord of *SOD1^G93A^* mice (Extended Data Fig. 2-2*A*,*C*). Therefore, to investigate the effect of the total Crmp1 amount, we analyzed *Crmp1*^−/−^/*SOD1^G93A^* mice and found that deleting *Crmp1* in *SOD1^G93A^* mice caused motor function to deteriorate slightly but did not affect survival or body weight. *Crmp1* knock-down was previously reported to reduce the number of spinal cord neurons *in vitro* (Kurnellas et al., 2010). In addition, *CRMP1* protein levels were reduced in the brains of HD patients, and those of *Crmp1* were decreased in a mouse model (Stroedicke et al., 2015). Moreover, CRMP1 knock-down by siRNA has been reported to enhance misfolding and toxicity of mutant huntingtin in an HD cell model, whereas CRMP1 overexpression shows the opposite effect (Stroedicke et al., 2015). These findings indicate that CRMP1 may have beneficial effects against neurodegenerative diseases, including HD and ALS, although the molecular mechanism of the decrease in CRMP1/Crmp1 in patients and mouse models is currently unknown. Unexpected mild deterioration of clinical (Fig. 2*F*) and pathologic (Fig. 3*A*,*D*) phenotypes in *Crmp1*^−/−^/*SOD1^G93A^* mice may reflect functional redundancy of other CRMP family proteins such as CRMP2.

We performed proteomics analysis followed by IPA analysis to investigate the underlying mechanism responsible for the phenotypic differences between *Crmp1*^−/−^/*SOD1^G93A^* and *Crmp1^ki^*^/^*^ki^*/*SOD1^G93A^* mice (Extended Data Fig. 3-1). The sirtuin signaling pathway was upregulated in *Crmp1^ki^*^/^*^ki^*/*SOD1*^G93A^ mice but downregulated in *Crmp1*^−/−^/*SOD1^G93A^* mice. Sirtuin signaling regulates cell survival, energy expenditure, and metabolic control through its energy-sensing and redox-sensing functions, and it is associated with lifespan extension (Tang, 2017). In ALS, the sirtuin signaling pathway has been reported to confer beneficial effects on motor neuron survival, such as promoting autophagy and mitophagy and suppressing protein misfolding and aggregate formation (Watanabe et al., 2014; Tang, 2017). These previous findings are consistent with the fact that the sirtuin signaling pathway reduces *SOD1*^G93A^ toxicity in the context of *Crmp1^ki^*^/^*^ki^* but increases it with *Crmp1*^−/−^. However, the mechanism of the relationship between Crmp1 alteration and the sirtuin signaling pathway in ALS model mice remains to be determined.

When considering therapeutic strategies, treatment effects may be enhanced by targeting not only the phosphorylation of CRMP1 but also that of CRMP2, because the inhibition of Crmp2 phosphorylation also ameliorates the motor phenotype of *SOD1^G93A^* mice (Numata-Uematsu et al., 2019). In humans, it is necessary to identify small-molecule inhibitors that selectively block Ser522 phosphorylation of CRMP1 and ideally also that of CRMP2. Alternatively, cell-permeable peptides that compete with CRMP1/2-Ser522 may be effective if used with appropriate cell-targeting vehicles to avoid the development of autoimmunity.

To conclude, we have comprehensively investigated proteins that are specifically phosphorylated in ALS model mice, and found evidence that Crmp1 phosphorylation at Ser522 is likely involved in ALS pathogenesis. Blocking Crmp1 phosphorylation at Ser522 led to improvements in the clinical and pathologic phenotypes of ALS model mice. These improvements were associated with alteration of the sirtuin signaling pathway. In humans, simultaneously suppressing Ser522 phosphorylation of both CRMP1 and CRMP2 may be a potential therapeutic strategy for ALS.

## Synthesis

Reviewing Editor: Laura Volpicelli-Daley, The University of Alabama at Birmingham Decisions are customarily a result of the Reviewing Editor and the peer reviewers coming together and discussing their recommendations until a consensus is reached. When revisions are invited, a fact-based synthesis statement explaining their decision and outlining what is needed to prepare a revision will be listed below. The following reviewer(s) agreed to reveal their identity: Krishna Aluri, Manuela Basso. Note: If this manuscript was transferred from JNeurosci and a decision was made to accept the manuscript without peer review, a brief statement to this effect will instead be what is listed below.

Both reviewers felt the authors did a satisfactory job addressing the previous concerns. Please do a spell check as mentioned by Reviewer 1.

Reviewer 1:

The authors present a case of involvement of Crmp1 phosphorylation in ALS G93A mice model. They used knockout and knockin mice to understand the role of crmp1 phosphorylation in extending the disease duration of the mice. the authors did a really good job to get a complete understanding of the role of phosphorylation and the pathways involved. I feel the authors sufficiently addressed previous reviewer comments.

Minor changes : phospho misspelled in line 446,449

Reviewer 2:

The authors addressed all my concerns. The manuscript is very well presented and of interest for the field.
