# Supplementary figures and images for: Inhibition of Crmp1 Phosphorylation at Ser522 Ameliorates Motor Function and Neuronal Pathology in Amyotrophic Lateral Sclerosis Model Mice
Source: eNeuro. 2022 May 23;9(3):ENEURO.0133-22.2022. doi: 10.1523/ENEURO.0133-22.2022 (PMC9131721; doi:10.1523/ENEURO.0133-22.2022)

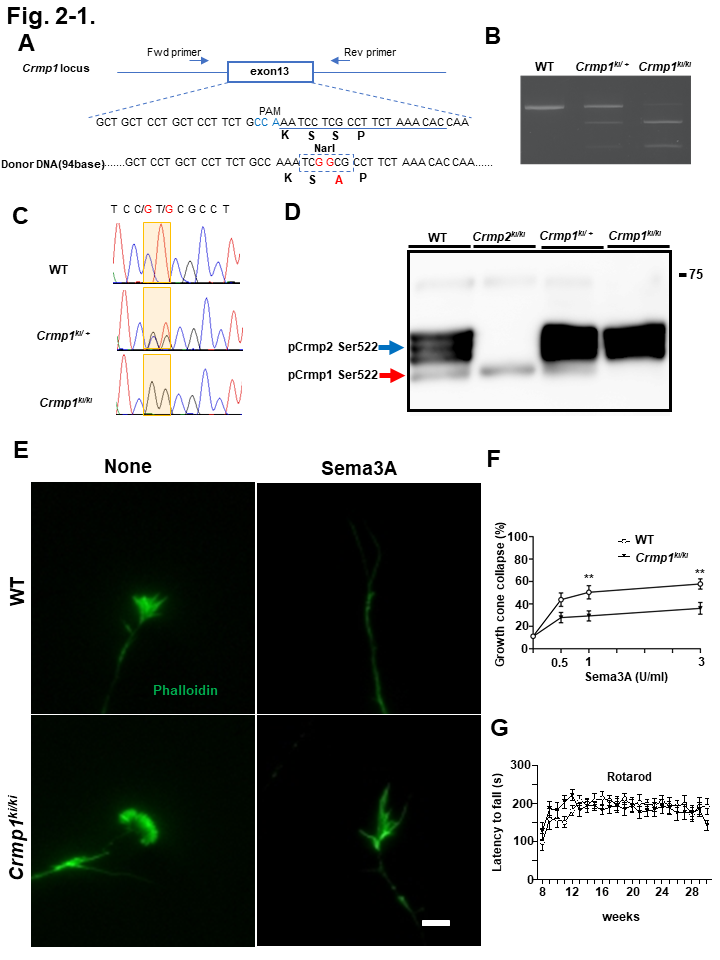

Supplement: Extended Data Figure 2-1 — Generation of Crmp1S522A knock-in mice. A, Substituted nucleotides and amino acids are shown as red letters. NarI restriction sites used for genotyping are shown as blue dotted squares. B, Genotyping analysis for mutated alleles by PCR. C, Sanger sequencing electropherograms for the targeted site in WT mice (top), heterozygous (middle), and homozygous knock-in (KI) offspring (bottom). Substitution sites in Crmp1 are indicated by square. D, Western blottings for phosphorylated Crmp1 and Crmp2 protein. Western blottings for WT, Crmp2 homozygous (ki/ki; nonphosphorylatable Crmp2S522A knock-in), Crmp1 heterozygous (ki/+), and homozygous (ki/ki) mouse brain lysates with anti-phospho-Crmp1/2-S522 antibody. Upper bands indicate phospho-Crmp2-S522 (blue arrow) and lower band indicates phospho-Crmp1S522 (red arrow). E, Morphology of E15 DRG growth cones of the WT and Crmp1ki/ki mice embryos treated with Sema3A. In WT DRG explants, Sema3A (1 U/ml) collapsed growth cones. By contrast, growth cone collapse induced by Sema3A was suppressed in Crmp1ki/ki DRG explants. Growth cones were stained with Alexa Fluor 488–phalloidin. Scale bar: 10 μm. F, SemaA concentration-response curve for growth cone collapse of WT and Crmp1ki/ki DRG neurons. Data are mean ± SEM for n = 8; **p < 0.01 by two-way ANOVA with Bonferroni’s multiple comparisons test. G, Accelerated rotarod performance (5–40 rpm/5 min) from 8 to 30 w. No significant differences were detected between WT and Crmp1 KI mice. Download Figure 2-1, TIF file. [file enu-eN-NWR-0133-22-s01.tif]

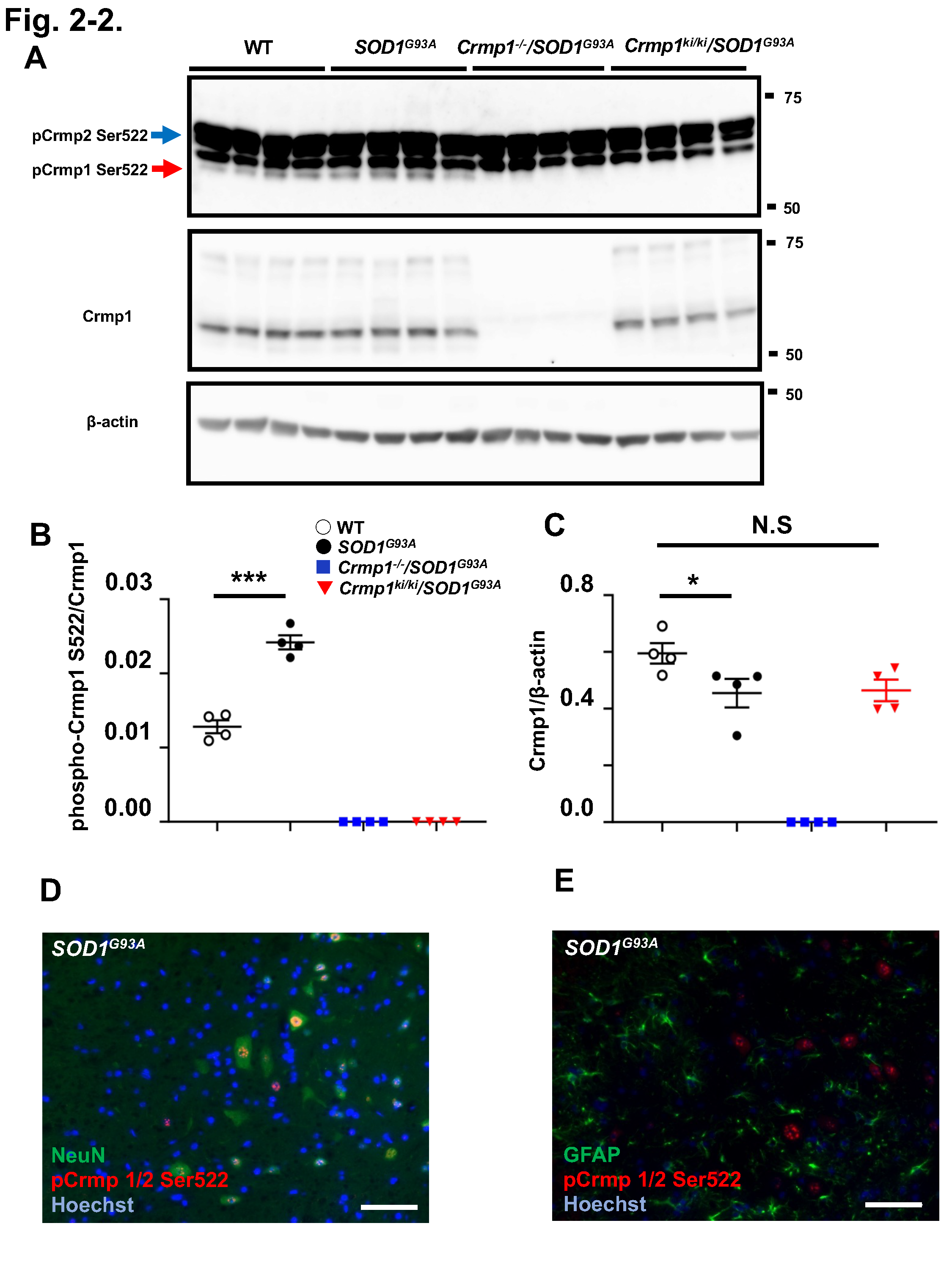

Supplement: Extended Data Figure 2-2 — Relative levels of phosphor-Crmp1-S522 and total Crmp1 in congenic SOD1G93A mouse strains. A, Western blottings for phospho-Crmp1-S522 and total Crmp1 protein level in the spinal cord of WT and SOD1G93A mutant mouse strains at 20 w. Upper bands correspond to phospho-Crmp2-S522 (blue arrow) while lower bands correspond to phospho-Crmp1-S522 (red arrow). B, The levels of phospho-Crmp1-S522 normalized to total Crmp1 are significantly higher in SOD1G93A mice than WT mice. ***p < 0.001 by unpaired t test. Statistical significance was determined as follows: WT versus SOD1G93A; p = 0.0001 by unpaired t test. C, Total Crmp1 protein was decreased in SOD1G93A mice compared with WT mice. *p < 0.05 by one-way ANOVA with uncorrected Fisher’s LSD. Statistical significance was determined as follows: WT versus SOD1G93A; p = 0.042, WT versus Crmp1ki/ki/SOD1G93A; p = 0.0549. D, Immunofluorescence data show that phospho-Crmp1/2-S522 (red) is co-localized with NeuN (green), as shown in the merged image (yellow) of the ventral horn of the lumbar spinal cord from SOD1G93A mice at 20 w. Nuclei were counterstained by Hoechst (blue). Scale bar: 50 μm. E, Phospho-Crmp1/2 S522 (red) and GFAP (green) were not colocalized in the ventral horn of lumbar spinal cord from SOD1G93A mice at 20 w. Nuclei were counterstained by Hoechst (blue). Scale bar: 50 μm. Download Figure 2-2, TIF file. [file enu-eN-NWR-0133-22-s02.tif]

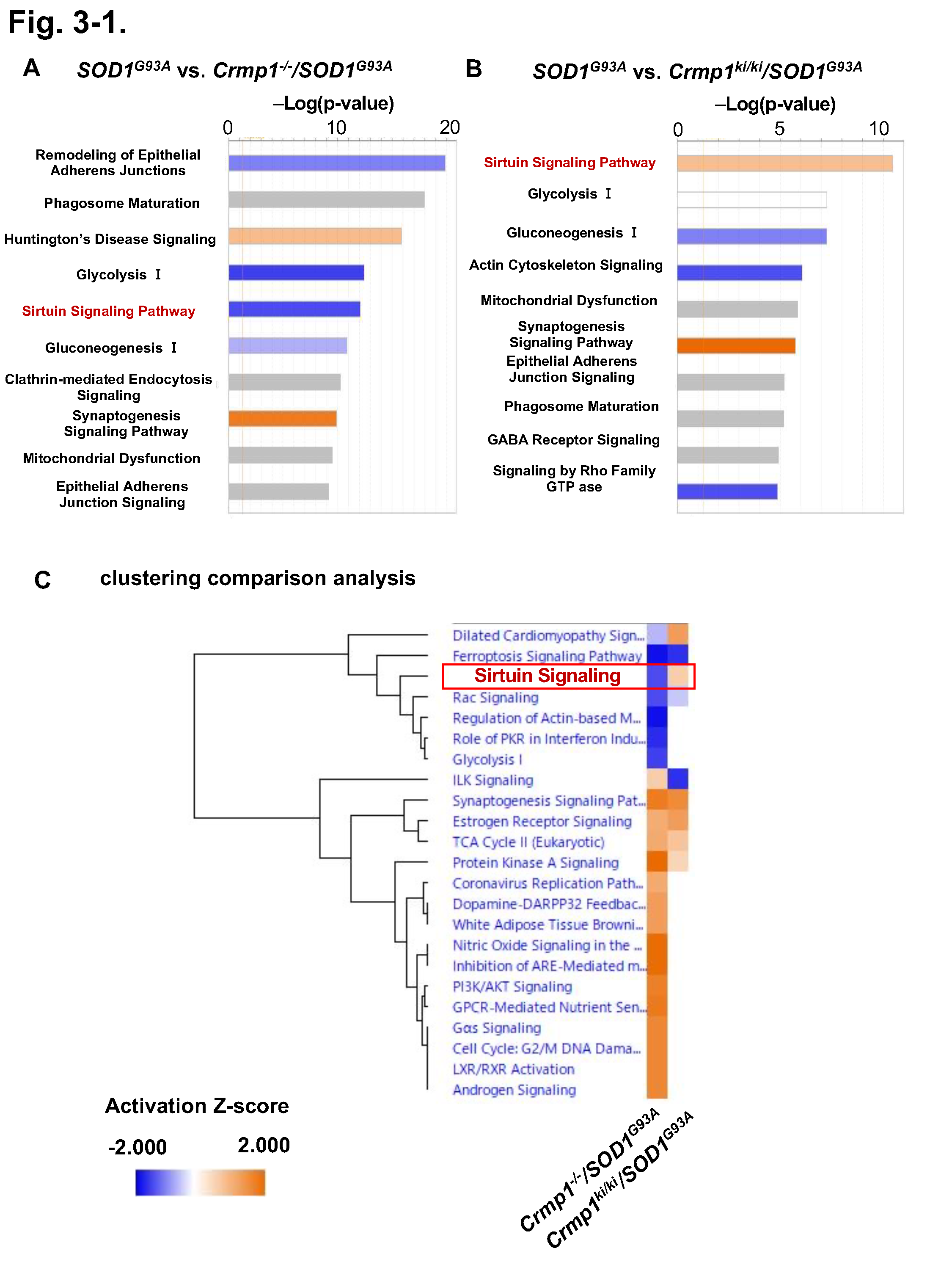

Supplement: Extended Data Figure 3-1 — Proteomic analysis and Western blotting of the spinal cord from SOD1G93A, Crmp1–/–/SOD1G93A, and Crmp1ki/ki/SOD1G93A mice. A, Top 10 canonical pathways identified by proteomics analysis of molecules differentially expressed (max fold change >1.5, ANOVA p < 0.05) between SOD1G93A and Crmp1–/–/SOD1G93A mice. B, Top 10 canonical pathways identified by proteomics analysis of molecules differentially expressed (max fold change >1.5, ANOVA with p < 0.05) between SOD1G93A and Crmp1ki/ki/SOD1G93A mice. C, Clustering analysis of differentially expressed canonical pathways between Crmp1–/–/SOD1G93A and Crmp1ki/ki/SOD1G93A mice. Download Figure 3-1, TIF file. [file enu-eN-NWR-0133-22-s03.tif]
